# Supplementary material for: Network heterogeneity regulates steering in actin-based motility
Source: Nat Commun. 2017 Sep 21;8:655. doi: 10.1038/s41467-017-00455-1 (PMC5608943; doi:10.1038/s41467-017-00455-1)
Supplement: Supplementary file 1 — Supplementary Information [file 41467_2017_455_MOESM1_ESM.pdf]

Description of Supplementary Files

File Name: Supplementary Information
Description: Supplementary Figures, Supplementary Methods and Supplementary References

File Name: Supplementary Movie 1
Description: **Growth of actin networks in the presence of Capping protein.** Reaction mixture: 6 µM G-actin Alexa-568 labeled, 18 µM Profilin, 120 nM Arp2/3 complex, 25 nM CP. Patterned geometries are GST-pWA-coated dot (left) and 3x15 μm^2 GST-pWA coated bar (right). Movie playback is 7 frames per seconds. Related to Figure 1.

File Name: Supplementary Movie 2
Description: **Growth of actin networks in the absence of Capping protein.** Reaction mixture: 1 µM G-actin Alexa-568 labeled, 3 µM Profilin, 80 nM Arp2/3 complex. Patterned geometries are 1x1 μm^2 GST-pWA-coated dot (left) and 3x15 μm^2 GST-pWA coated bar (right). Movie playback is 7 frames per seconds. Related to Figure 1.

File Name: Supplementary Movie 3
Description: **Growth of actin networks on adjacent nucleation sites**. Reaction mixture: 6 µM G-actin-Alexa-568 labeled, 18 µM Profilin, 120 nM Arp2/3 complex, 25 nM CP. Patterned geometries are twin bars of 3 x 15 μm^2 spaced by 25 µm (left) or 6 µm (right). Movie playback is 7 frames per seconds. Related to Figure 2.

File Name: Supplementary Movie 4
Description: **Growth of actin networks of different sizes.** Reaction mixture: 6 µM G-actin Alexa-568 labeled, 18 µM Profilin, 120 nM Arp2/3 complex, 25 nM CP. Patterned geometries are bars of 3 x 15 μm^2, 3 x 30 μm^2, and 3 x 90 μm^2 (from left to right). Movie playback is 7 frames per seconds. Related to Figure 2.

File Name: Supplementary Movie 5
Description: **Laser patterning of Polystyrene coated surface.** The printed patterns consisted of matrices of spots of adjustable density. Left (4.8 spots. μm^{-2}), middle (1.4 spots. μm^{-2}) and right (0.66 spots. μm^{-2}). Movie playback is 42 frames per seconds. Related to Figure 3.

File Name: Supplementary Movie 6
Description: **Growth of actin networks of different densities: same pattern organization (same number of spots) but different nucleation promoting factor densities.** Reaction mixture: 6 µM Gactin-Alexa-568 labeled, 18 µM Profilin, 120 nM Arp2/3 complex, 25 nM CP. Pattern organization is 2.5 spots. μm^{-2}. Three different densities of nucleation promoting factor on patterns were obtained using 0.4, 0.5, and 0.6 µM GST-pWA during the coating (from left to right). Movie playback is 7 frames per seconds. Related to Figure 4.

File Name: Supplementary Movie 7
Description: **Growth of actin networks of different densities: different pattern organizations (different number of spots) but the same nucleation promoting factor density by spot.** Reaction mixture: 6 µM G-actin Alexa-568 labeled, 18 µM Profilin, 120 nM Arp2/3 complex, 25 nM CP. Pattern organization is 2.5 (left) and 6.6 spots. μm^{-2} (right). The same density of nucleation promoting factor on patterns was obtained using 0.5 µM GST-pWA during the coating. Movie playback is 7 frames per seconds. Related to Figure 5.

File Name: Supplementary Movie 8
Description: **Steering the growth of heterogeneous actin networks toward higher densities (left).** Reaction mixture: 6 µM G-actin Alexa-568 labeled, 18 µM Profilin, 120 nM Arp2/3 complex, 25 nM CP. Pattern organization is 2.5 spots. μm^{-2}. Two densities of nucleation promoting factor per pattern: each half of the pattern was coated with 0.6 µM or 0.3 µM GST-pWA. Movie playback is 7 frames per seconds. Related to Figure 6B. **Steering the growth of heterogeneous actin networks toward lower densities (right).** Reaction mixture: 6 µM G-actin Alexa-568 labeled, 18 µM Profilin, 120 nM Arp2/3 complex, 25 nM CP. The pattern organization is mixt: one half has 6.6 and the other half has 2.5 spot. μm^{-2}. The same density of nucleation promoting factor on patterns was obtained using 0.5 µM GST-pWA during the coating. Movie playback is 7 frames per seconds. Related to Figure 6C.

File Name: Supplementary Movie 9
Description: **Steering the growth of gradient of heterogeneous actin networks toward lower densities.** Reaction mixture: 6 µM G-actin Alexa-568 labeled, 18 µM Profilin, 120 nM Arp2/3 complex, 25 nM CP. The pattern organization has a gradually decreasing density from 8.3 to 2 spots. μm^{-2} every 3x5 μm^2. The same density of nucleation promoting factor on patterns was obtained using 0.5 µM GST-pWA during the coating. Movie playback is 7 frames per seconds. Related to Figure 6F (left).

File Name: Peer Review File

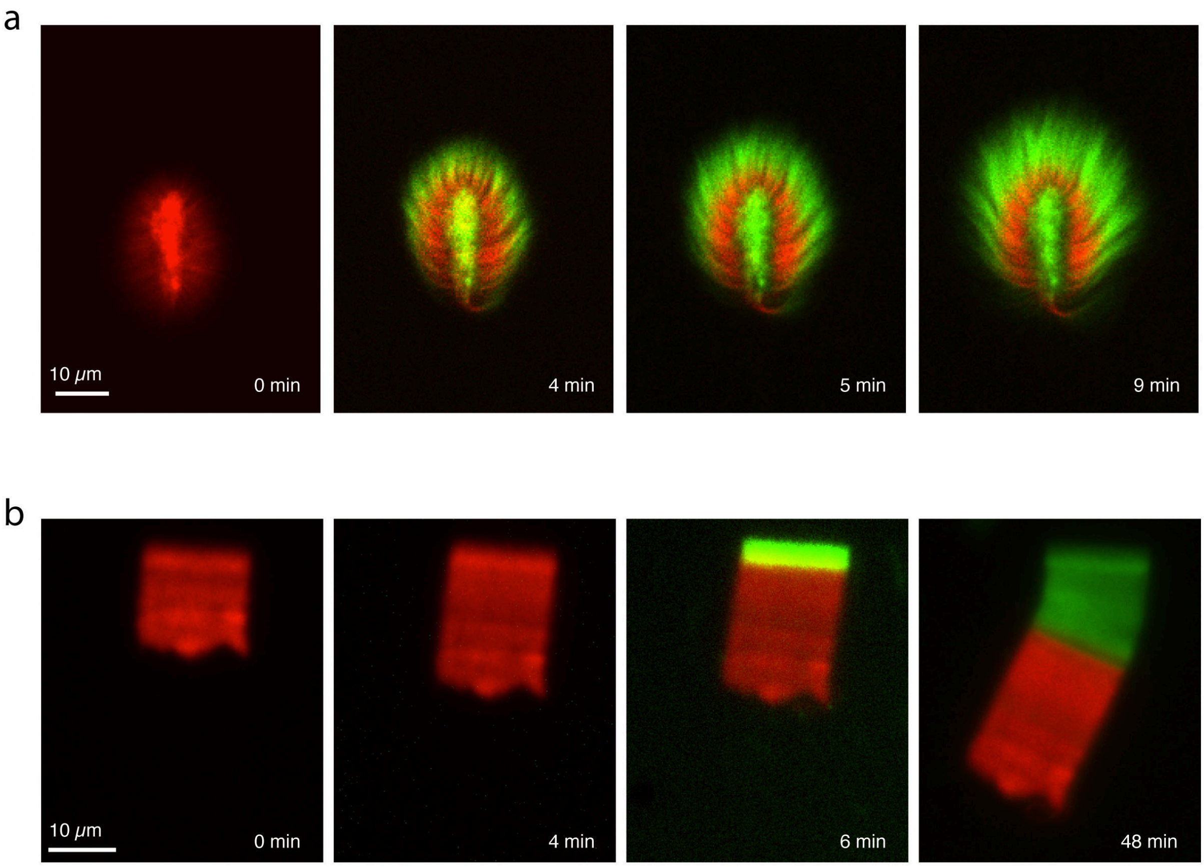

Supplementary Figure 1: Addition of capping protein induces a structural reorganization of the actin network. Actin networks were assembled on NPFs-coated 3x15 μm bars in a flow chamber of 10 μm height. Sequential flows of Alexa568 labeled (red) actin then Alexa488 (green) actin containing polymerization medium were performed. **(a)** In the absence of capping protein, and in the presence of 1 μM actin (10% labeled), 3 μM profilin, and 80 nM Arp2/3 complex, green actin was added to parallel bundles growing out of the bar (elongation) and was incorporated into the branched network on the bar (nucleation and elongation). **(b)** In the presence of capping protein, and in the standard conditions of the motility reconstitution assays (see methods), addition of green actin was constrained to the vicinity of the nucleation bar (nucleation and elongation).

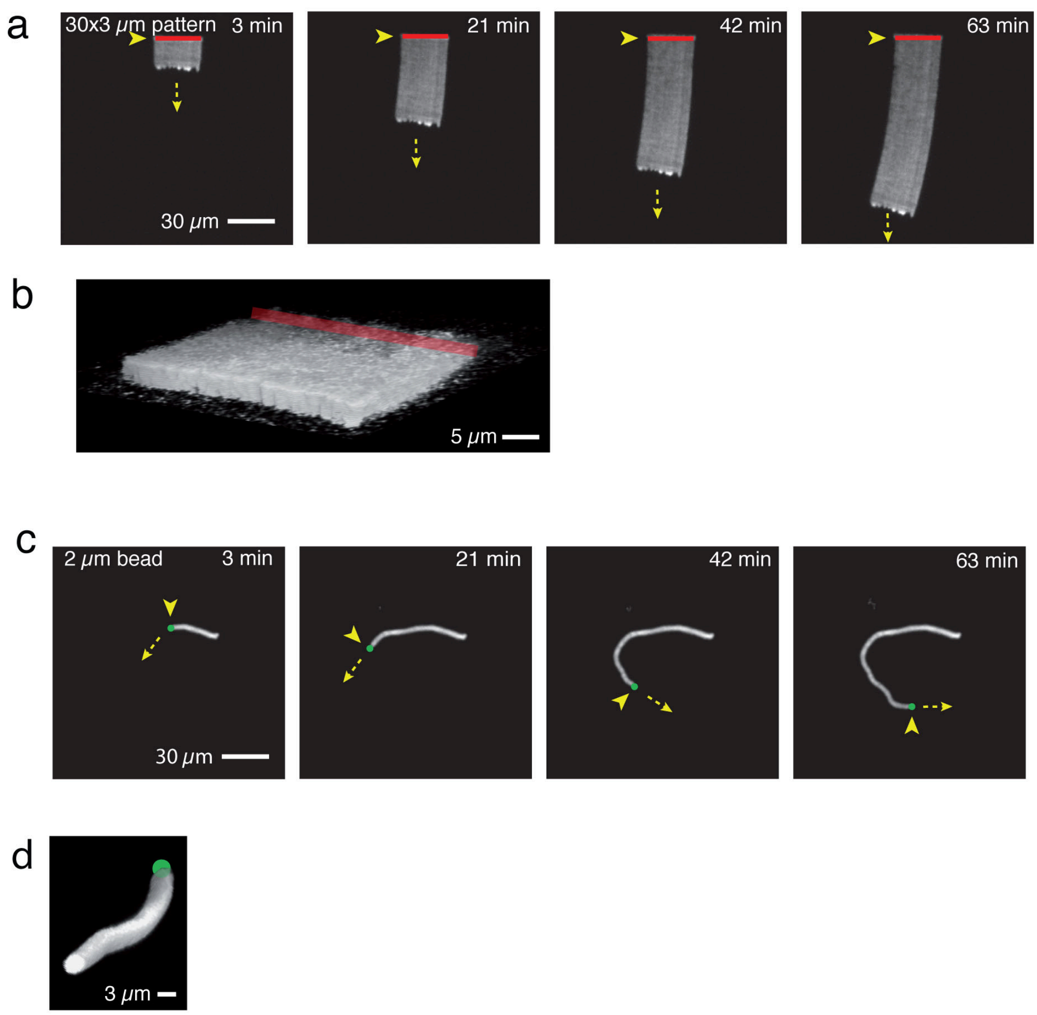

Supplementary Figure 2: Comparison between reconstituted LMs and standard bead motility assay. (a) 2D-growth of lamellipodium-like actin structure. LMs were reconstituted on functionalized NPFs-coated bar-shaped patterns (3x30 μm^2). **(b)** Three-dimensional reconstruction of the confocal images in **(a)** showing the flat and thin section of lamellipodium-like actin structure. **(c)** Actin-based motility was reconstituted on functionalized NPFs-coated polystyrene beads (4,5 μm in diameter). **(d)** Three-dimensional reconstruction of the confocal images in **(c)**, showing the cylindrical section of actin comet tails polymerized on beads **(a-d)** Arrowheads indicate the nucleation site, arrows the forward (beads) or rearward (LMs) propulsion, green dot the bead, red bar the pattern.

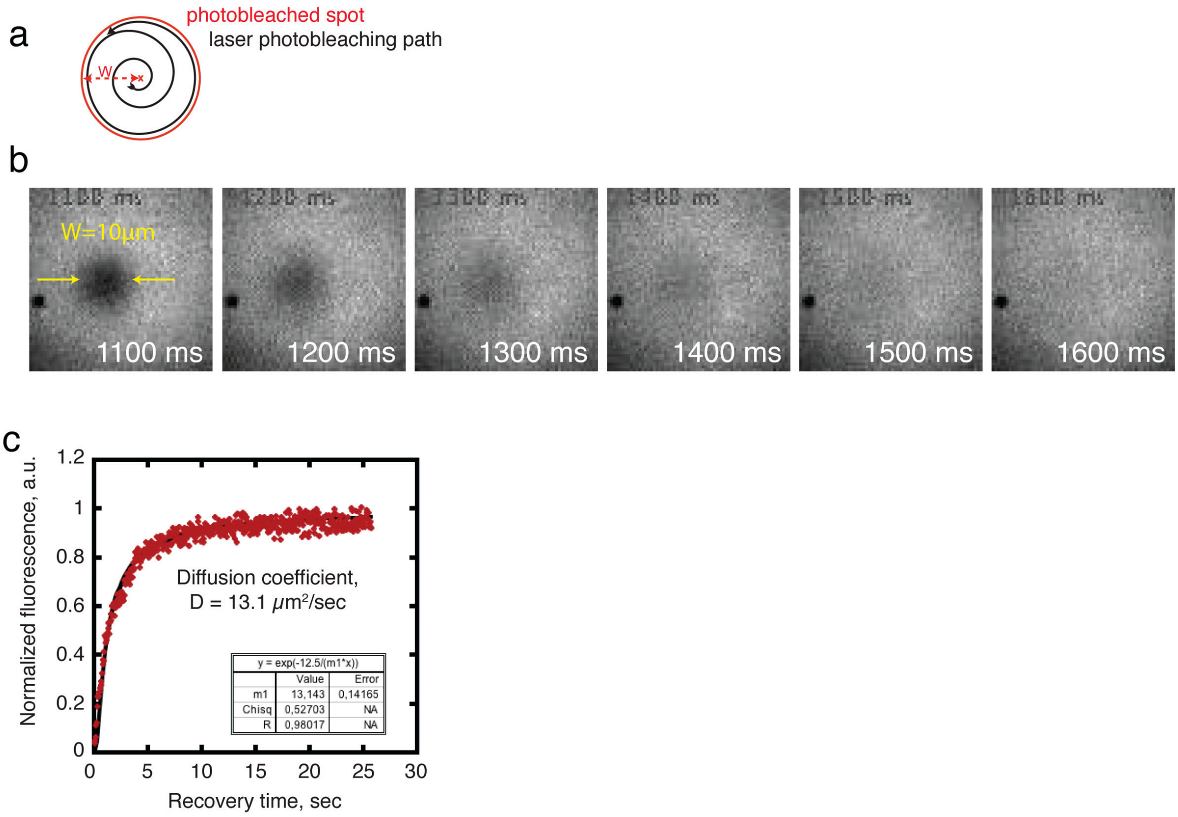

Supplementary Figure 3: Measurement of the experimental diffusion coefficient during reconstitution assays of LMs. (a) Schematic showing the spiral laser path, from the center to the periphery to photobleach a cylinder of 10 µm in radius. **(b)** Timelapse images of the fluorescence recovery followed in an XY cross section of the cylinder using a confocal microscopy. Fluorescent monomers were exchanged from the periphery of a disk and monomer exchange in the Z direction was negligible. Thus, we considered the fluorescence recovery of a photobleached disk and **(c)** the diffusion coefficient was extracted from the equation derived by Soumpasis:

$f(t) = \exp(-2\tau_D/t).[I_0(2\tau_D/t)+I_1(2\tau_D/t)]$ where $2\tau_D=w^2/(4D)$, f(t) is the normalized fluorescence that goes to 1 as t goes to infinity, I_0 and I_1 are Bessel functions, t is the time, and τ_D is the characteristic time scale for diffusion. τ_D was computed for a bleached spot (disk) of a radius w and a diffusion coefficient D. The data were fitted with Bessel functions to the order zero.

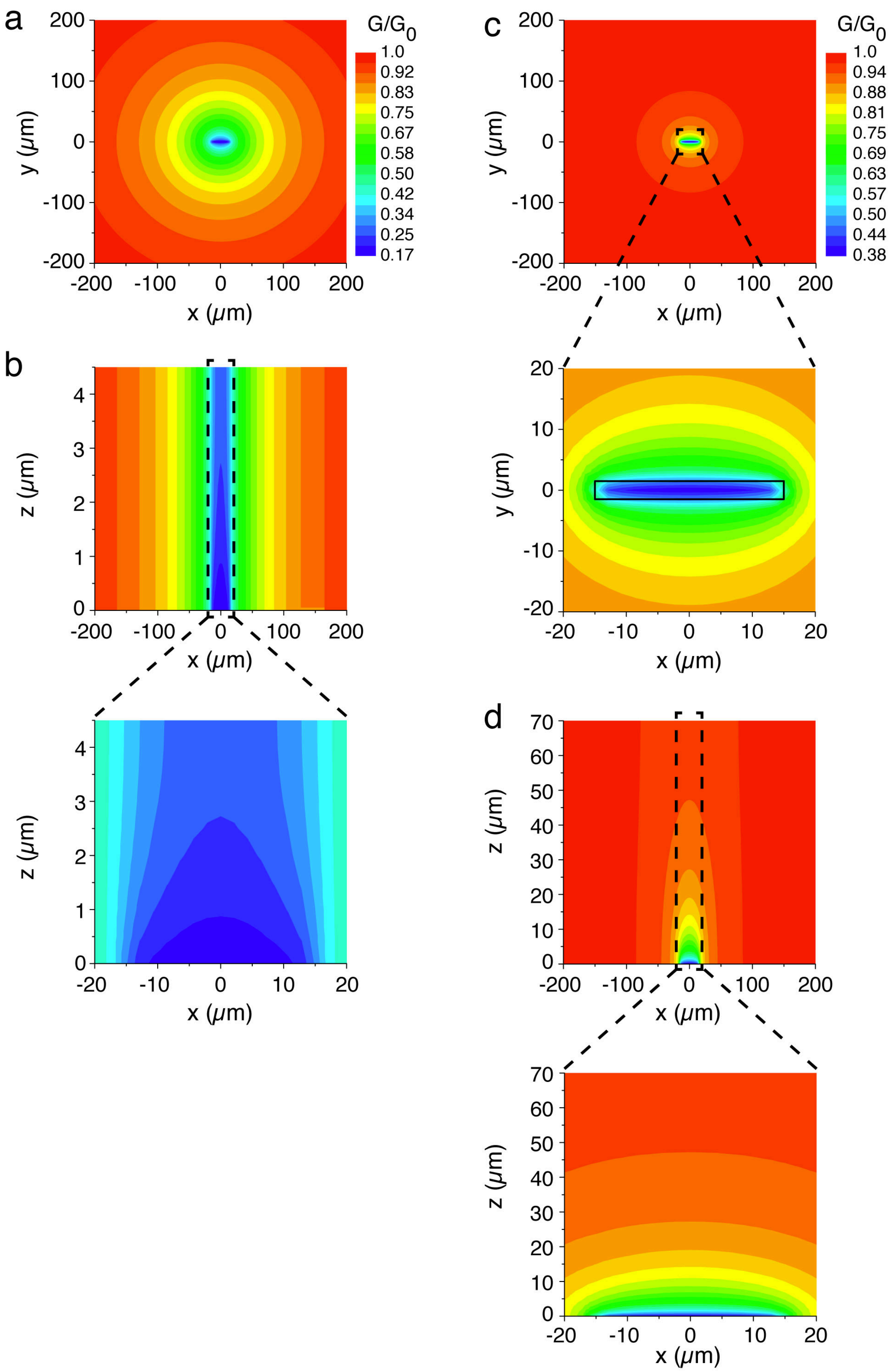

a
b
c
d
G/G0
1.0
0.92
0.83
0.75
0.67
0.58
0.50
0.42
0.34
0.25
0.17
0.94
0.88
0.81
0.69
0.63
0.57
0.44
0.38
x (μm)
y (μm)
z (μm)

Supplementary Figure 4: Quantitative modeling of the local monomers depletion effect at the nucleation site during LMs growth. (a, b) Computed distribution of the G-actin concentration in the "2D" case (4 μm-deep chamber). Monomer density at 200×200 μm^2 area around the nucleation site (**a**) and in the cross section of the chamber perpendicular to the plane of the nucleation site (**b**) are shown.

(c, d) Same in the "3D" case (70 μm-deep chamber). The concentration is shown in units of G-actin concentration far away (mm scale) from the nucleation sites. Zooms show areas near the nucleation sites where the monomer depletion is especially rapid. Note the quantitative differences between the "2D" and "3D" cases: in 3D, the gradient of G-actin near the nucleation site is steeper, therefore more monomers are delivered by diffusion, and the local G-actin concentration is higher in 3D.

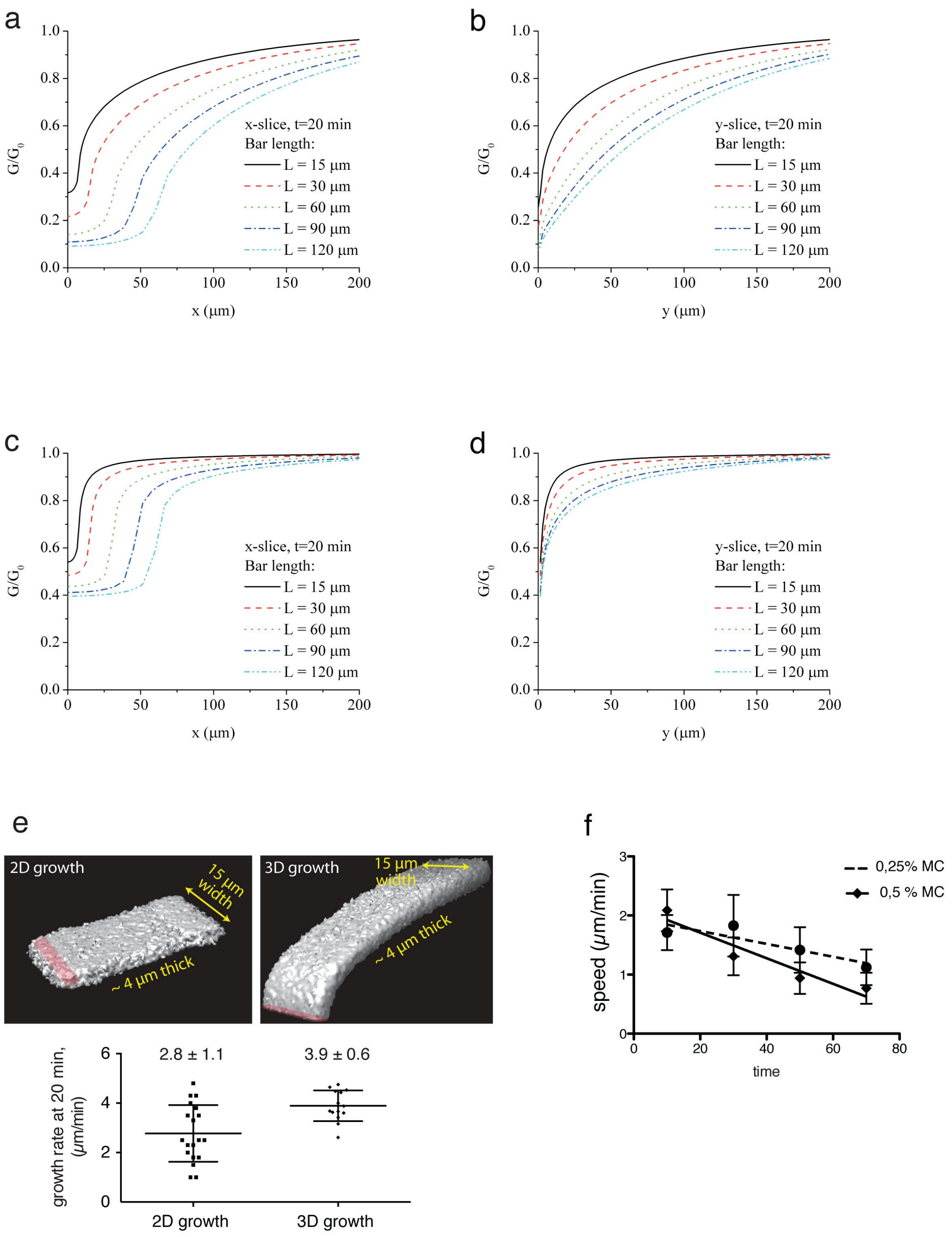

Supplementary Figure 5: Simulated depletion effect shows a dependency on the size of the actin nucleation pattern and on the dimensions of the G-actin volume. (a-d) Computed G-actin concentration as a function of the coordinate, for patterns of width 3 µm and variable lengths, along the line through the center of the

nucleation area, parallel to the long side of the rectangular nucleation domain **(a,c)** and normal to it **(b,d)**. **(a,b)** and **(c,d)** correspond to the "2D" and "3D" cases, respectively. The result for t = 20 min is shown, with global G-actin concentration $G_0 = 6\mu M$, density of growing filaments at the leading edge $\rho = 300/\mu m^2$, and geometry/force factor $\Phi = 0.7$. **(e)** In agreement with mathematical simulations, the growth rate is statistically slower in a small (2D growth) than in a large (3D-growth) polymerization condition. The images are three-dimensional reconstruction of the confocal images of LMs polymerized as indicated on 3x15 μm^2 bar-shaped patterns (red bars). Error bars show mean s.d. for n=19 (2D growth), n=12 (3D-growth) LMs per condition. **(f)** 2D-Growth rate of LMs polymerized on 3x15 µm2 was measured over time for 2 concentrations of methylcellulose. Increasing the percentage of methylcellulose in the reconstitution medium lowers the diffusion coefficient, reducing therefore LMs growth rate. This is in agreement with the existence of diffusive gradients of monomers set by actin assembly at the nucleation site.

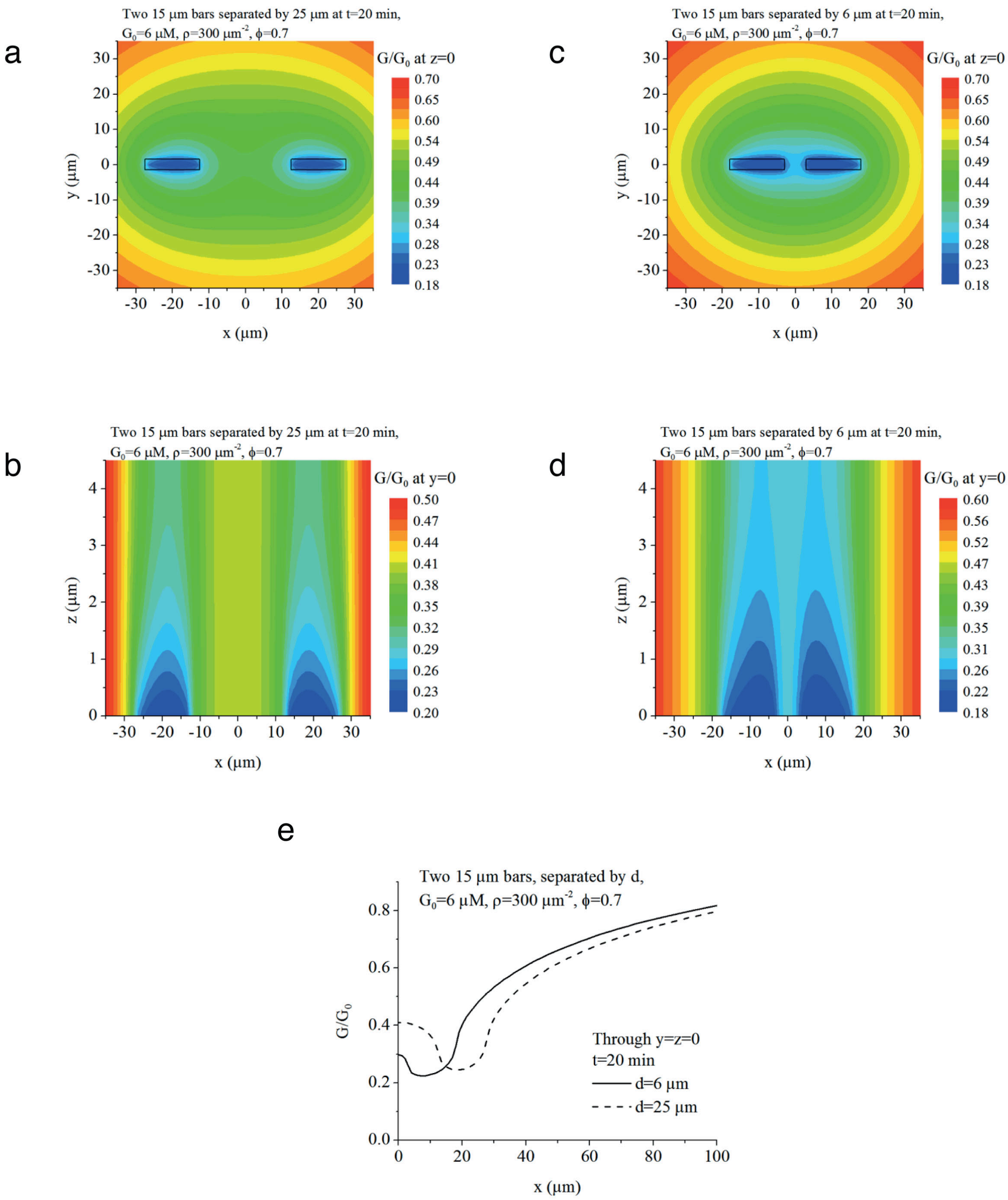

Supplementary Figure 6: Simulated depletion effect is additive in case of adjacent nucleation sites. (a-d) Computed G-actin concentration in the "2D" case for two rectangular 15 µm long nucleation patterns the contours of which are outlined in black in **(a,c)**. Concentration is shown as function of 2-D coordinates in the plane of the 'bottom' of the experimental chamber, where the nucleation patterns are **(a,c)**,

and in the perpendicular plane through the long axes of these patterns **(b,d)**. **(a,b)** and **(c,d)** correspond to 25 μm and 6 μm distances between the nucleation domains, respectively. The concentrations are color-coded; parameters are the same as in Supplementary Figure 5. **(e)** The same G-actin concentrations are shown along the line through the center of the nucleation patterns, parallel to their long axes.

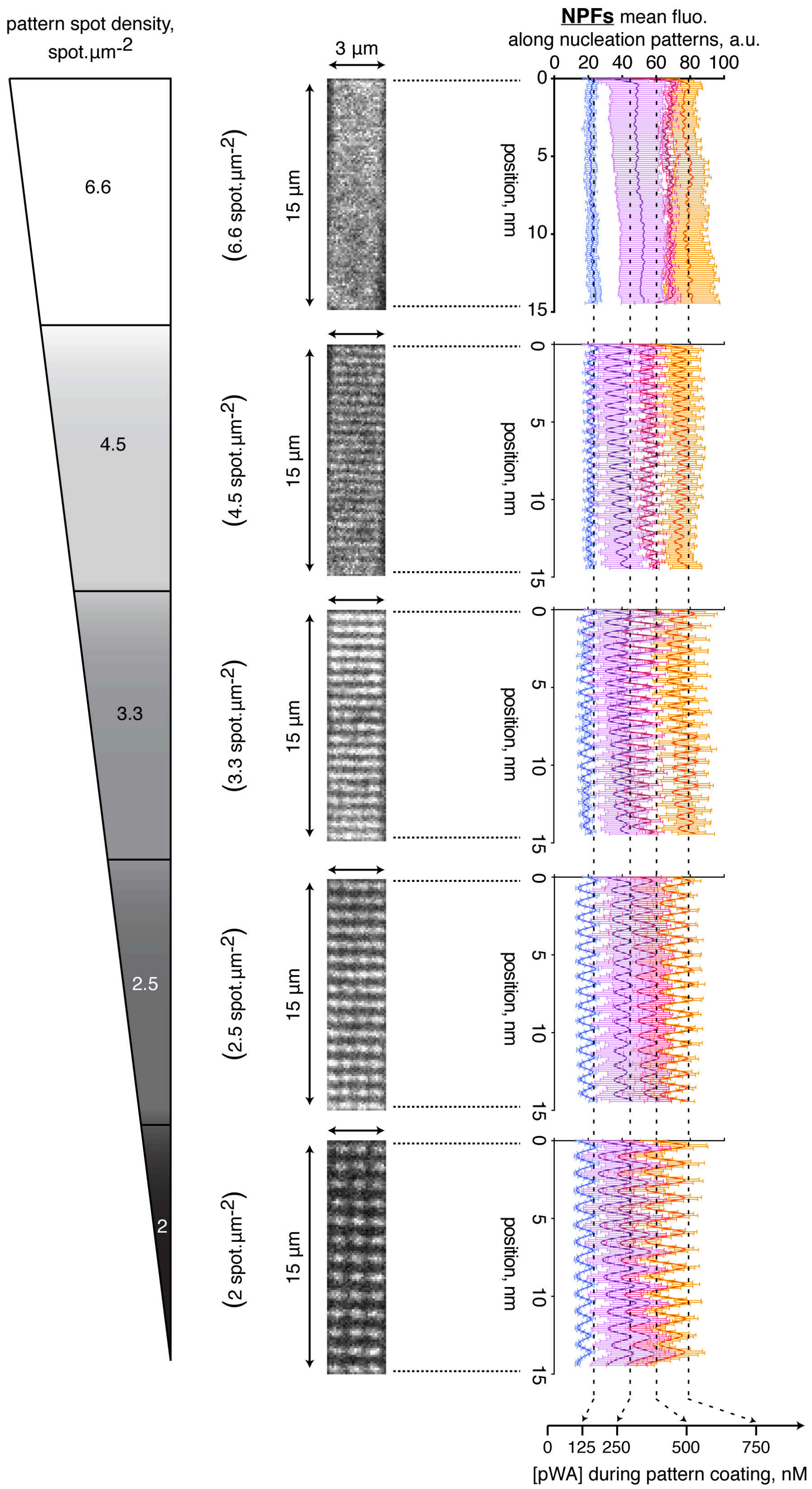

pattern spot density,
spot.µm-2
6.6
4.5
3.3
2.5
2
(6.6 spot.µm-2)
(4.5 spot.µm-2)
(3.3 spot.µm-2)
(2.5 spot.µm-2)
(2 spot.µm-2)
3 µm
15 µm
NPFs mean fluo.
along nucleation patterns, a.u.
0 20 40 60 80 100
position, nm
0 5 10 15
0 125 250 500 750
[pWA] during pattern coating, nM

Supplementary Figure 7: Laser patterning is a reliable method to accurately control the NPFs density and organization at the nucleation sites. The printed patterns consisted of matrices of spots of adjustable density. We limited our investigations to densities ranging from 6.6 to 2 spots/μm^2; the latter was the experimental limit to reconstitute cohesive and continuous LMs sheets. To assess the reproducibility of the patterning procedure, several patterns of different spot densities were printed on the same coverslip, and several coverslips were coated with different concentrations of Alexa488-NPFs, as indicated. The analysis accurately showed (i) internal (throughout the patterning on the same coverslip) and external (comparing patterning on different coverslips) invariability of the spot density as we obtained a perfect superimposition of the sinusoidal curves, and (ii) the NPFs density per laser spot was invariable for each spot density as well as for the different spot densities, and this remains true for each concentration of NPFs used during the pattern coating (alignment of the maxima of sinusoidal curves along the doted lines).

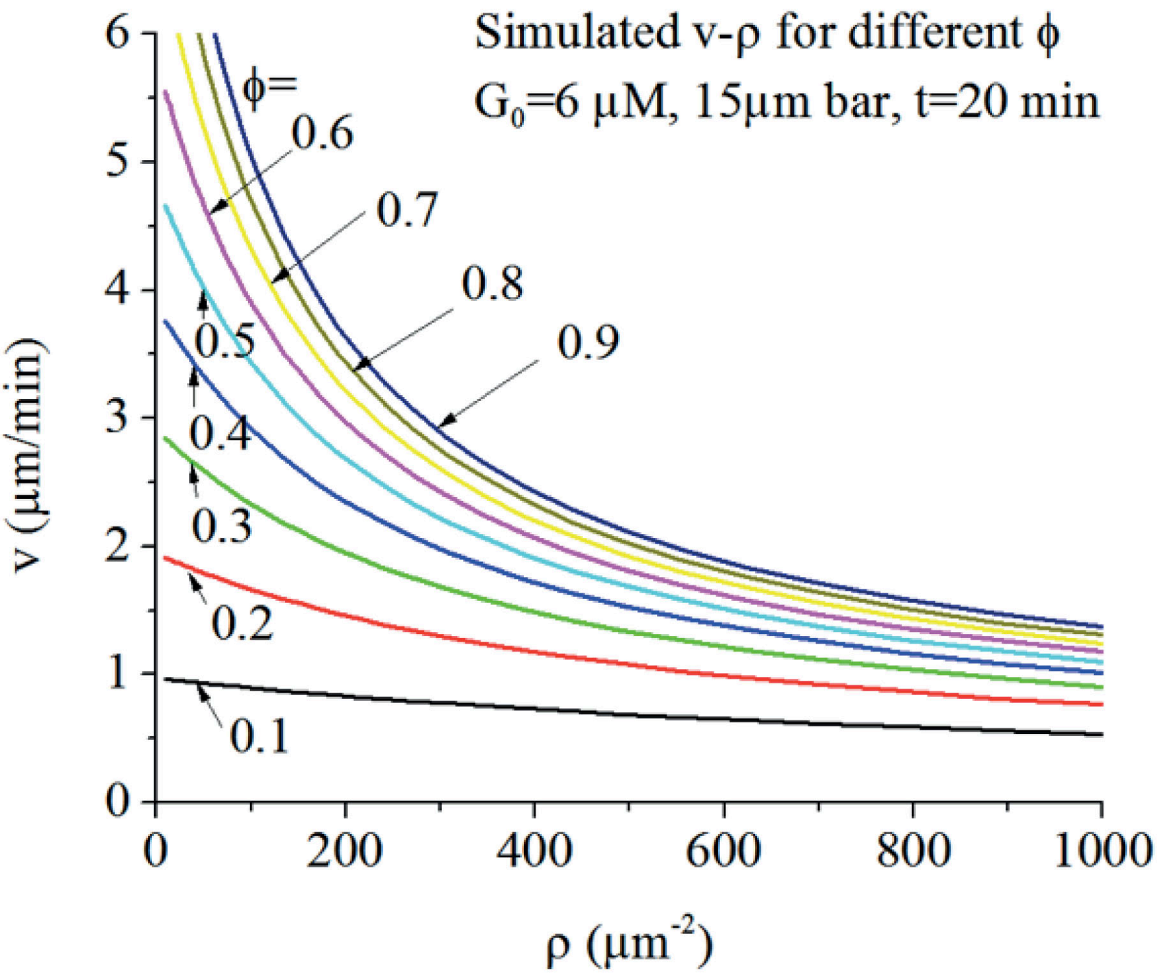

Supplementary Figure 8: Simulated growth rate of LM depends both on the geometric factor and the local monomer depletion. Predicted rate of growth of actin network from the 15×3 μm rectangular nucleation pattern in the "2D" case for varying geometry/force factor Φ. The rates are plotted as functions of the density of growing filaments at the leading edge. The results for t = 20 min are shown, with global G-actin concentration $G_0 = 6\mu M$.

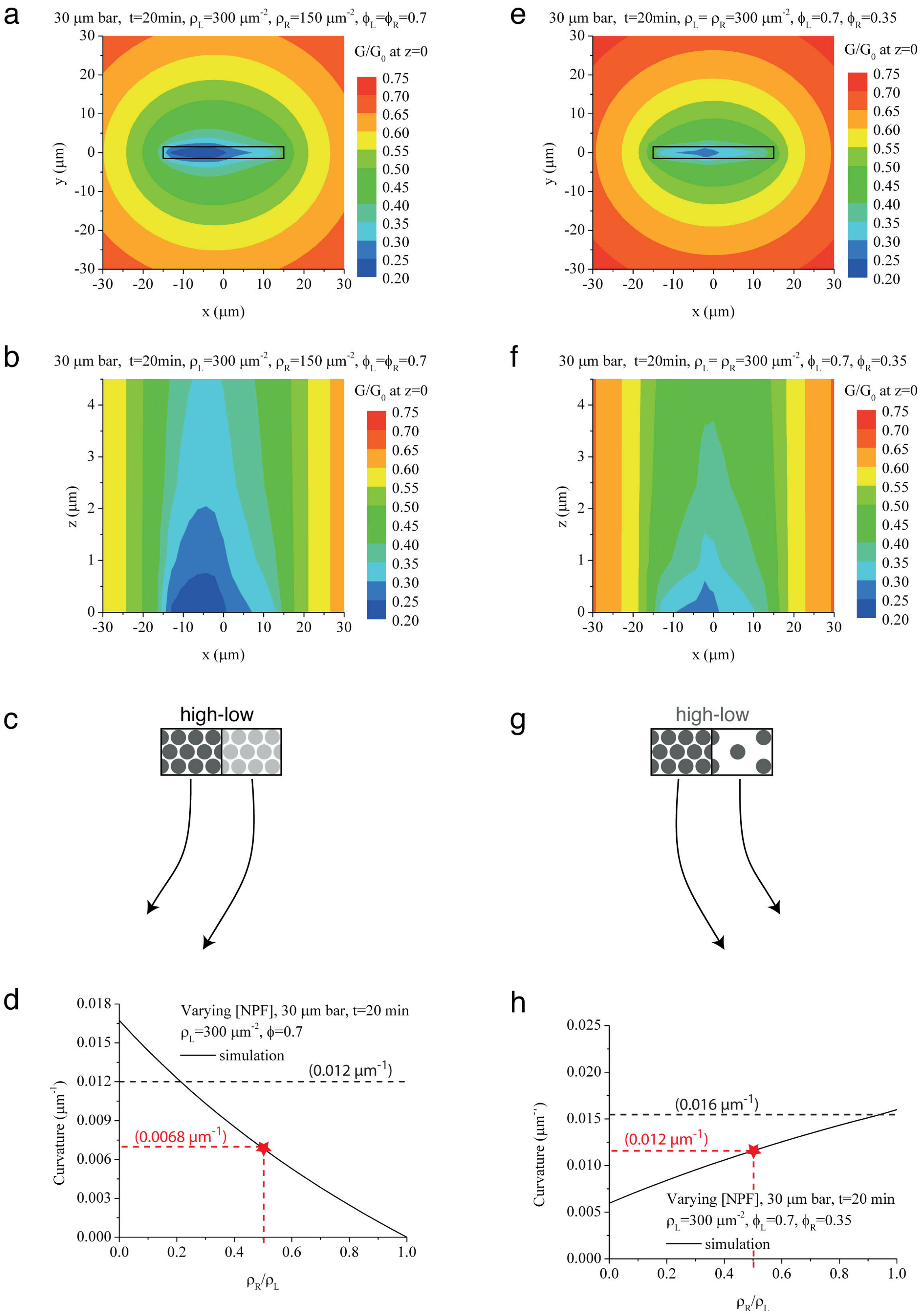

Supplementary Figure 9: Simulations predict the steering of the heterogeneous networks. (a-b, e-f) Computed G-actin concentration in the "2D" case with heterogeneous rectangular 30 μm long nucleation patterns. All results are for

t = 20 min, with global G-actin concentration $G_0 = 6\mu M$. Concentration is shown as function of 2-D coordinates in the plane of the 'bottom' of the experimental chamber, where the nucleation patterns are **(a,e)**, and in the perpendicular plane through the long axes of these patterns **(b,f)**. The concentrations are color-coded **(a-b)** In this case, corresponding to geometry **(c)**, each half of the nucleation domain has different actin density, $\rho_L = 300/\mu m^2$ at the left, and $\rho_R = 150/\mu m^2$ at the right. The geometric/mechanical factor $\Phi = 0.7$ is the same for both halves. **(e-f)** In this case, corresponding to geometry **(g)**, each half of the nucleation domain has different NPF distribution, and thus different geometric/mechanical factors, $\Phi_L = 0.7$ at the left, and $\Phi_R = 0.35$ at the right. The actin density $\rho = 300/\mu m^2$ is the same for both halves. **(c,g)** Two different geometries of NPF distributions. **(d)** Predicted curvature of the actin network corresponding to the nucleation geometry **(c)** as function of the right/left ratio of the actin densities (solid curve); Red star represents the computed value. The dashed line corresponds to the average experimentally measure curvature. **(h)** Same as **(d)** corresponding to the nucleation geometry **(g)**.

Supplementary Methods

Mathematical supplement

Estimate for the speed of actin network growth:

Free (unloaded) growth speed of the actin network is $V_0 = k_{on}\delta G$, where $k_{on} \approx 10/\mu M \cdot s$ is the polymerization rate, $\delta \approx 0.003\mu m$ is the half-size of actin monomer, and *G* is the local G-actin concentration at the leading (growing) edge of the network[1,2]. Considering that the observed speed is $0.03\mu m/s$, the local G-actin concentration at the leading edge is $1\mu M$, six-fold lower than the initial G-actin concentration $6\mu M$. Three explanations are possible: global depletion of monomers over time, slowing down of actin growth by mechanical load, and the local depletion. Below, we consider all three factors.

The monomers in the chamber are not globally depleted over time:

Assuming even dense actin network, with average distance, *d*, between the neighboring growing barbed ends very small, equal to 30 nm[3], there are $3\mu m \times 30\mu m/d^2 \quad 10^5$ filaments at the leading edge of the network growing at the $3\mu m \times 30\mu m$ nucleation domain. Considering that the 'actin tail' grows to $100\mu m$ in length, the total length of F-actin is $10^7\mu m$, and it contains $10^7\mu m/0.003\mu m \quad 3 \cdot 10^9$ monomers. There are about 600 molecules in one cubic micron of a solution with $1\mu M$ concentration[2], to reflect that, we will use parameter $\omega \approx 600/\left(\mu M \cdot \mu m^3\right)$. The volume of the chamber is $W \approx 20mm \times 20mm \times 4.5\mu m = 2 \cdot 10^9 \mu m^3$, so the chamber contains $\omega \times W \times 6\mu M \approx 10^{13}$ monomers, orders of magnitude more than 'consumed' by

the network, so the decrease of the network growth speed is not the result of the global actin depletion.

Note about the Arp2/3 complex and capping protein:

It is easy to demonstrate that neither Arp2/3 complexes, nor capping protein are depleted globally over the time of the experiment. Indeed, considering that there are one Arp2/3 complex and one capping protein per filament, and using the estimate of the number of actin filaments from above, there are $3\mu m \times 30\mu m \times 100\mu m / d^3 \quad 10^8$ filaments in total, and so the same number of Arp2/3 complexes and capping protein will be consumed. The initial concentrations of Arp2/3 and capping protein are 100 and 20 nM, respectively. These concentrations correspond to ~ $\omega \times W \times 0.1\mu M \approx 1.7 \times 10^{11}$ and ~ $\omega \times W \times 0.02\mu M \approx 3.3 \times 10^{10}$ molecules of Arp2/3 and capping protein, respectively, which is more than two orders of magnitude more than the consumed amount. Therefore, these concentrations decrease by less than one per cent, which is negligible. Considering that these proteins have to be delivered by diffusion to the leading edge of the growing actin network, we have to estimate whether the diffusion flux can deliver them. The total diffusive flux (see below) is of the order of $\frac{2\pi}{3} DR\omega P$ where D is the diffusion coefficient, $R \sim 6$ µm is the leading-edge size, and P is the diffusing protein concentration. Considering that Arp2/3 complex is a few-fold greater than actin monomer, if we assume that its diffusion coefficient is $D \quad 10\mu m^2 / s$, its flux could be of the order of 10^5 molecules per second, and this is exactly how much is needed. Indeed, there are a few thousand filaments at the leading edge produced per second, with one Arp2/3 per filament. Similarly, as capping protein is a few folds smaller than actin monomer, if we assume that its

diffusion coefficient is $D \quad 50 \mu m^2 / s$, its flux could be, again, of the order of 10^5 molecules per second, and this is exactly how much is needed, as there is one capping protein per filament.

Mechanical load on the growing actin network:

The rough estimate for the upper limit of the viscous resistance for the growing lamellipodial network in the “2D” experiment is the shear stress between the chamber wall and creeping network, $\eta V / l$, where η is the viscosity, *V* is the growth speed, and l is the distance between the flat network and chamber wall, multiplied by the area of the lamellipodial network, $A \quad 3 \cdot 10^3 \mu m^2$ [4]. Taking viscosity a few-fold that of water, $\eta \quad 3 \cdot 10^{-3} pN \cdot s / \mu m^2$, and the distance between the flat network and chamber wall as small as $l \sim 0.01 \mu m$, we have the force $\eta VA / l$ of the order of tens of *pN* distributed over $30 \mu m$ of the leading edge. The resulting $1 pN / \mu m$ is certainly not enough to slow down the network growth. In “3D” the viscous resistance to the growing pillar of actin is of the order of $10 \eta VL \sim 0.1 pN$, where $L \sim 100 \mu m$, also negligible, even in the presence of methylcellulose that can increase the viscosity several orders of magnitude. Resistance to Darcy flow through the porous actin meshwork is not likely to create related force in both “2D” and ”3D” experiments, because the fluid likely moves with the growing network.

In addition, in the “2D” experiment, the actin filaments of the lamellipodial sheet could, in principle, generate friction by repeated bending and straightening on microscopic bumps on the chamber wall. This force could be significant: as one bent filament could exert a pN-level force[5], tens of thousands of bent filaments at the sides of the actin network in contact with the walls can create sizeable load of the

order of hundreds of $pN/\mu m$ at the leading edge. As the lamellipodial length increases with time, the number of pushing filaments at the leading edge does not change, while the actin network elongates increasing the contact with the wall, which could be part of the observed decrease of the growth speed with time. However, such force is absent in the “3D” experiment, and a small difference between the growth rates in the “2D” and ”3D” experiments can be fully quantitatively explained by the effect of the local monomer depletion, so we propose that the friction between the actin and the walls generate a negligible load because of smoothness of the wall.

G-actin concentration is locally depleted at the leading edge:

Here we estimate local depletion of monomers in simplified 2D model; below, we solve the equations in the exact 3D geometry. The actin monomer distribution in the experimental chamber is governed by the equation:

$$\frac{\partial G}{\partial t} = D\left(\frac{\partial^2 G}{\partial x^2} + \frac{\partial^2 G}{\partial y^2}\right) - \alpha G \cdot \Omega(x,y), \qquad [1]$$

where *D* is the diffusion coefficient, and Ω is equal to 1 on the nucleation domain (for $-L/2 < x < L/2, -h/2 < y < h/2$) and to 0 otherwise. For the rough estimate, we neglect the density gradients over the shallow depth of the chamber. The monomer consumption rate α can be estimated as follows: d^{-2} filaments are growing per square micron of the pattern (we are using $d = 50$ nm [3]). This growth consumes $V/\delta d^2$ monomers per second, which translates into $V/\left(\delta d^2 \omega H\right)$ micromolar per second. Using the formula for the growth speed, $V = k_{on}\delta G\Phi$ where Φ is the factor decreasing the speed due to the geometry and load force, we have $\alpha = k_{on}\Phi / d^2\omega H = \alpha_0\Phi \approx (1.5/\sec)\Phi \approx 1/\sec$ (see below for the estimate of factor Φ).

This is a significant consumption: let G_l be the G-actin concentration at the leading edge, and G_0 - in the chamber far away from the growing network. Then, $\alpha G_l Lh$ G-actin is consumed at the pattern, and this G-actin is brought by the diffusive flux: $\alpha G_l Lh \quad D\left(G_0 - G_l\right)$.

Then, we can estimate:

$$G_l \quad \frac{D}{D+\alpha Lh} G_0 \qquad [2]$$

Diffusion coefficient is $D \approx 13 \mu m^2/s$ (Figure S3); $\alpha Lh \quad 50-100 \mu m^2/s$ (for $L = 15-30 \mu m$), so estimated $G_l \quad \frac{1}{5} G_0 - \frac{1}{9} G_0$. The observed actin growth rate corresponds to $G_l \quad \frac{1}{6} G_0$ indicating that the modeling explanation of the slowing growth down by the effect of the local G-actin depletion is correct. Formula [2] also predicts that the local G-actin, and hence speed, will decrease with the length of the pattern, as well as when diffusion decreases (methylcellulose is used), as observed.

Estimate of factor Φ :

If the size of the nucleation domain is below $1 \mu m^2$, then expression $\alpha Lh < 2 \mu m^2/s << D$ and for actin networks that small, the local depletion of monomeric actin is negligible. In this case, the slowdown of the actin growth, according to the model, is due to the geometric/mechanical factor Φ only. From the results reported in Fig. 1a,b, $\Phi \approx 0.7$.

Difference between "2D" and "3D" cases:

Rough analytical estimates allow to understand the difference between "2D" and "3D" cases. In the 2D, we can solve analytically stationary diffusion equation [1] outside the disc-like nucleation domain with radius R, so that the boundary condition at the disc boundary is monomeric concentration being equal to G_l, and at larger domain boundary with radius $\tilde{R}$ ($\tilde{R}$ is the radius of the area from which monomers are depleted), monomeric concentration being equal to G_0. In this case[4] the flux of monomers into the nucleation domain is equal to:

$$J_{2D} = \frac{2\pi}{\ln\left(\tilde{R}/R\right)} DH\omega\left(G_0 - G_l\right)$$

[3]

In the 3D case, for $\tilde{R} >> R$, such flux is equal to[4]:

$$J_{3D} = \frac{2\pi}{3} DR\omega\left(G_0 - G_l\right)$$

Here $H = 4.5\mu m$ is the height of the experimental chamber. For the nucleation domain area $A = 100\mu m^2, R \approx 5.6\mu m$ is the effective radius of the pattern ($A = \pi R^2$). In the 2D case, the simplest way to estimate radius $\tilde{R}$ is to use the formula[4]

$\tilde{R} = 2\sqrt{Dt}$ for the distance of expansion by diffusion of the perturbation to the monomeric distribution by the nucleation domain that starts at time $t = 0$. Note that for $t = 20\min$ this formula predicts $\tilde{R} \approx 250\mu m$ which is in excellent agreement with the numerical simulations. Equalizing the consumption rates and flows of monomers in 2D and 3D, we have:

2D: $G_l = \dfrac{D}{D + \alpha_2} G_0, \alpha_2 = \dfrac{k_{on}\Phi A \ln\left(\tilde{R}/R\right)}{2\pi H\omega d^2}$

3D: $G_l = \frac{D}{D+\alpha_3} G_0, \alpha_3 = \frac{2k_{on}\Phi R}{3\omega d^2}$

Here *d* is the average distance between actin filaments. Finally, substituting these expressions into the formula for the growth rate, we have:

2D: $V_2 = V_0 \frac{\Phi}{1+\chi_2 n\Phi}, \chi_2 = \frac{k_{on} A \ln\left(\tilde{R}/R\right)}{2\pi H\omega D d_0^2}, \tilde{R} = 2\sqrt{Dt}$ [4]

3D: $V_3 = V_0 \frac{\Phi}{1+\chi_3 n\Phi}, \chi_3 = \frac{2k_{on} R}{3\omega D d_0^{\ 2}}$ [5]

Here $n = d_0^{\ 2}/d^2$ is the non-dimensionalized F-actin density.

We are using formulas [4, 5] in the main text to evaluate the rate of growth of actin networks as functions of actin density and factor Φ. Using the model parameters, we estimate $\chi_2 \approx 3.8$, and $\chi_3 \approx 1.6$. Thus, theory predicts that in 3D the depletion of monomers is lower because the diffusion flux supplies monomer from greater volume. Interestingly, the model also predicts that in 2D the local monomer concentration decreases with time because monomers are depleted from greater and greater volume, and diffusive gradient and flux become weaker gradually. In contrast, in 3D, the flux is time-independent. Indeed, measurements show fast and slow decrease of the actin growth rate with time in 2D and 3D, respectively.

Numerical results:

We solve numerically equation:

$\frac{\partial G}{\partial t} = D\left(\frac{\partial^2 G}{\partial x^2} + \frac{\partial^2 G}{\partial y^2} + \frac{\partial^2 G}{\partial z^2}\right)$, [6]

with the following boundary conditions: diffusive flux is equal to zero at the boundaries of the 3D volume, except on the nucleation domain on the 'floor' of the

volume. At the nucleation domain, the diffusive flux of monomers into the boundary is equal to $\alpha G(x, y, z = 0)$ with coefficients α estimated above. The dimensions of the computational domain are: length and width are 500 μm, more than the region from which monomers are depleted over 30 min, and the height of the domain is equal to the height of the used experimental chamber in “2D” and ”3D” cases. The initial condition is a constant concentration equal to G_0.

The results of these calculations for rectangular nucleation domains are shown in (supplementary Fig.4 and supplementary Fig. 5a-d, 8a-b, e-f). The results for two nucleation domains at two different distances from each other are shown in supplemental figure S6. To obtain the predictions shown in main (Fig. 2f,g), the results of the calculations of the G-actin concentration for the rectangular nucleation domain in “2D” and ”3D” cases, respectively, were substituted into the expression for the actin growth rate. We varied the filament density near the nucleation domain and showed the result in supplementary Fig. 8. By using a constant conversion coefficient between actual density and the actin fluorescence signal that gave the best fit between the measurements and predictions, we plotted main Fig. 4c and 5d.

To calculate the curvature of the ‘actin tail’, we solve the diffusion equation for G-actin for the rectangular domain two halves of which are characterized by different actin densities and factors Φ. The results are shown in supplementary Fig. 8. Then, we compute the average rates of the actin growth V_l and V_r at the left and right halves, respectively, and estimate the radius of curvature R using the following geometric argument: as the angular speed of actin tail turning is the same at the left and at the right of the actin network, then $(R+l)/V_l = (R-l)/V_r$ where l is the half-length of the

nucleation domain. Thus, the curvature $\frac{1}{R}=\frac{1}{l}\frac{V_l-V_r}{V_l+V_r}$. The results are shown in main Fig. 6g and supplementary Fig.9.

Relevance of the results to motile cells

We use modeling in the following paragraph to show that the actin monomer depletion effect is relevant for the lamellipodial leading edge. Let us consider the geometry of a generic lamellipodium which can be approximated with a rectangular parallelepiped with a broad and wide rectangular base in the XY-plane being the ventral surface, and narrow leading edge of height h in the YZ-plane. Roughly, there is little variance in any relevant density in Y and Z directions, and so we can approximately consider G-actin density as function of just distance from the leading edge X and time T. Assuming for simplicity that all actin assembly is at the leading edge, while the disassembly is uniformly spread throughout the lamellipodium, equation for G-actin distribution has the form:

$$\frac{\partial G}{\partial T}=D\frac{\partial^2 G}{\partial X^2}+S, \frac{\partial G}{\partial X}|_{X=L}=0, D\frac{\partial G}{\partial X}|_{X=0}=\tilde{\alpha}G(0)$$

Here S is the G-actin source from F-actin disassembly, L is the front-to-end lamellipodial size, and assembly factor $\tilde{\alpha}$ relates to the factor α estimated above as follows: $\tilde{\alpha}=\alpha H$ (based on comparison of the derivation above and (2)), providing that the mesh size of the network is the same, and in all known *in vitro* and *in vivo* cases it is of the order of a few tens of nanometers, the in vivo and in vitro situations are governed by the same scales. Importantly, note that in this 1D model parameter α does not depend on the lamellipodium height h: the monomer 'consumption' scales with the lamellipodial height h, but so does the diffusive flux. Thus, the balance of the flux and monomer consumption is independent of the lamellipodial thickness: in the

in vitro experiments, the network is ~ 10-20 times thicker than characteristic lamellipodium, but the diffusive flux is also an order of magnitude greater than that in thin lamellipodium. If we scale the G-actin equation as follows: $X = Lx, T = \frac{L^2}{D}t, G = \frac{L^2 S}{D}g$, the non-dimensional equation becomes:

$$\frac{\partial g}{\partial t} = \frac{\partial^2 g}{\partial x^2} + 1, \frac{\partial g}{\partial x}|_{x=1} = 0, \frac{\partial g}{\partial x}|_{x=0} = \left(\frac{\tilde{\alpha} L}{D}\right) g(0)$$

It has the solution:

$$g = \frac{D}{\tilde{\alpha} L} + x - \frac{x^2}{2}$$

The depletion effect is pronounced as far as the non-dimensional parameter $\frac{D}{\tilde{\alpha} L} < 1$, or $\frac{D}{\alpha H L} < 1$. We have $\frac{D}{\alpha H L} \sim \frac{13 \mu m^2 / s}{1/s \times 4\mu m \times 10 \mu m} \sim \frac{1}{3}$, and so there will be a significant depletion in the lamellipodium.

In order to investigate potential role of a great concentration of thymosin-sequestered monomers in the depletion effect, let us consider the following model in the same lamellipodial geometry:

$$\frac{\partial G}{\partial T} = D\frac{\partial^2 G}{\partial X^2} + k_1 \tilde{G} - k_2 G, \frac{\partial G}{\partial X}|_{X=L} = 0, D\frac{\partial G}{\partial X}|_{X=0} = \tilde{\alpha} G(0)$$
$$\frac{\partial \tilde{G}}{\partial T} = D\frac{\partial^2 \tilde{G}}{\partial X^2} - k_1 \tilde{G} + k_2 G + S, \frac{\partial \tilde{G}}{\partial X}|_{X=L} = 0, D\frac{\partial \tilde{G}}{\partial X}|_{X=0} = 0$$

The additional assumptions here are: the disassembly source increases the population of the sequestered monomers $\tilde{G}$ (because thymosin exchanges with cofilin on ADP-G-actin), and then there is an exchange between the pools of sequestered and non-sequestered monomers with the rates k_1, k_2 (the order of magnitude of which

is 1/s (2)). With the same rescaling as above, the system of equations becomes:

$$\frac{\partial g}{\partial t}=\frac{\partial^2 g}{\partial x^2}+\left(\frac{k_1L^2}{D}\right)\tilde{g}-\left(\frac{k_2L^2}{D}\right)g,\frac{\partial g}{\partial x}|_{x=1}=0,\frac{\partial g}{\partial x}|_{x=0}=\left(\frac{\tilde{\alpha}L}{D}\right)g(0)$$

$$\frac{\partial \tilde{g}}{\partial t}=\frac{\partial^2 \tilde{g}}{\partial x^2}-\left(\frac{k_1L^2}{D}\right)\tilde{g}+\left(\frac{k_2L^2}{D}\right)g+1,\frac{\partial \tilde{g}}{\partial x}|_{x=1}=0,\frac{\partial \tilde{g}}{\partial x}|_{x=0}=0$$

Assuming that the average concentration of the sequestered monomers is significantly greater than that of non-sequestered monomers, and due to the no-flux boundary conditions for the sequestered monomers, $\tilde{g}\approx const$, and the effective equation for the non-sequestered monomers has the form:

$$\frac{\partial g}{\partial t}=\frac{\partial^2 g}{\partial x^2}+C-\left(\frac{k_2L^2}{D}\right)g,\frac{\partial g}{\partial x}|_{x=1}=0,\frac{\partial g}{\partial x}|_{x=0}=\left(\frac{\tilde{\alpha}L}{D}\right)g(0)$$

where C is a constant. The solution for this equation is:

$$g(x)\approx\bar{g}\left[1+\frac{\alpha H}{\sqrt{Dk_2}}\left(1-\exp\left[-\sqrt{\frac{k_2}{D}}Lx\right]\right)\right]$$

The depletion effect is significant as far as the factor $\frac{\alpha H}{\sqrt{Dk_2}}$ is not much smaller than 1. We have $\frac{\alpha H}{\sqrt{Dk_2}}\sim\frac{1/s\times 4\mu m}{\sqrt{13\mu m^2/s\times 1/s}}\sim 1$, and so the depletion effect still accompanies the situation with greater overall g-actin concentrations in motile cells.

Note that the monomer concentration in our *in vitro* experiments is ~ 6 μM, which is likely the same as the concentration of polymerizable monomers in cells, because this concentration produces the polymerization rate of the order of that observed *in vivo* and of the same order that we observed in our *in vitro* experiments. It is likely, that total concentration of monomers in cells is higher, and that the major fraction is sequestered and unavailable for polymerization. Calculations above demonstrate that

all our qualitative conclusions in that case remain the same. We are unaware of quantitative measurements of NPF density in motile cells, but what matter is, in fact, the actin network density (or mesh size). To measure it quantitatively in vitro would require either EM or calibrated fluorescent TIRF microscopy, which is beyond the scope of our study. However, all available *in vivo* and *in vitro* data in the literature indicates that functional actin networks have mesh size of the order of tens of nanometers. Smaller mesh size likely renders filaments inflexible, unable to generate much force; more importantly, the monomer depletion effect would so great that there would be no actin growth. Larger mesh size corresponds to such long and disentangled filaments that they would buckle rather than grow. Therefore, it is likely that the F-actin density in our *in vitro* experiments is of the same order of magnitude as that in the cells.

Regarding possible mechanisms behind the observed effect of the network's growth rate decreasing with growing inhomogeneity of the NPF distribution, largely speaking, two, not mutually exclusive, effects can explain this effect. First, there could be that average filament orientation in the network changes, so that the angle between the leading filaments and the surface they push on could become smaller (filaments grow more parallel to the surface in the more inhomogeneous networks). This could happen because filaments generated at the NPF spots bend or turn to reach and fill the spaces between the spots. In this case, the same rate of elongation of individual filaments translates into a slower growth of the network's leading edge. Another mechanistic reason for this change in the network architecture is that the network is a weaved mesh of narrow actin tails originating at the NPF spots, and when the NPF spots are sparse, the tails buckle and meander, effectively decreasing the angle

between the leading filaments and the surface they push on, slowing the protrusion down. Second, there could be a change of mechanical balance between the pushing and tethered filaments, for example, relatively more filaments get tethered between the NPF spots, the mechanical resistance to protrusion increases, and the network growth slows down. Yet another possibility is that larger spaces between the NPF spots could lead to lesser filaments' entanglement, which makes the actin network more deformable, so that it recoils under load and protrudes slower.

Our observation that the actin network growth rate increases with decreasing NPF concentration, providing that the character of the NPF distribution does not change, is likely to be limited to actin networks of physiological-range densities. For a very low density network, the growth rate will stop increasing for at least three reasons. First, at a density approximately an order of magnitude lower than that in the *in vitro* experiments, the monomer depletion effect becomes negligible, and the growth rate becomes independent from the NPF concentration. Second, if the external mechanical load is not scaling with the number of pushing filaments, at low F-actin density such load would overwhelm the network growth mechanically. Third, by the law of large numbers, spatial inhomogeneity increases when the network density decreases, and as we showed this would lead to slower protrusion of the low-density networks.

How relevant is the turning mechanism that we propose to the turning of cells *in vivo*? The mechanism of cell turning is largely unclear, and there are likely multiple mechanisms, in fact. Some cells turn by generating new front and rear after extinguishing pre-existent front and rear[6,7], others turn harnessing waves of

protrusion-retraction[8,9]. On the other hand, cells with broad and steady lamellipodial fronts, like keratocytes[10,11], nerve growth cones[12], fibroblasts under certain conditions[13], and neutrophils (famous video on the internet of a crawling neutrophil chasing a bacterium made in the 1950s by the late David Rogers at Vanderbilt University) can turn by pivoting their lamellipodial fronts, and in those cases the mechanism we are proposing is feasible. In fact, the role of G-actin concentration for orienting the leading edge was highlighted in[12], while the role of NPF for the leading edge turning was proven in[11].

Data and code availability

All relevant data are available from the authors.

Numerical codes used to solve the reaction-diffusion equations describing actin monomer distributions can be downloaded from:

http://cims.nyu.edu/~mogilner/codes.html

Supplementary References:

1. Pollard, T.D., Blanchoin, L., and Mullins, R.D. Molecular Mechanisms Controlling Actin Filament Dynamics in Nonmuscle Cells. *Annu. Rev. Biophys.* **29**, 545-576. (2000).
2. Mogilner, A., and Edelstein-Keshet, L. Regulation of actin dynamics in rapidly moving cells: a quantitative analysis. *Biophys. J.* **83,** 1237-1258 (2002).
3. Abraham, V.C., Krishnamurthi, V., Taylor, D.L., and Lanni, F. The actin-based nanomachine at the leading edge of migrating cells. *Biophys. J.* **77,** 1721-1732 (1999).
4. Berg, H.C. Random walks in biology. Princeton Univ. Ress, Princeton. (1983).
5. Mogilner, A., and Oster, G. The physics of lamellipodial protrusion. *Eur. Biophys. J.* **25,** 47-53(1996).
6. Sugawara M, Miyoshi H, Miura T, Tanaka H, Tsubota KI, Liu H. Dynamics of actin stress fibers and focal adhesions during slow migration in Swiss 3T3 fibroblasts: intracellular mechanism of cell turning. *Biomed. Res. Int.* 2016:5749749 (2016).
7. Leyman S, Sidani M, Ritsma L, Waterschoot D, Eddy R, Dewitte D, Debeir O, Decaestecker C, Vandekerckhove J, van Rheenen J, Ampe C, Condeelis J, Van Troys M. Unbalancing the phosphatidylinositol-4,5-bisphosphate-cofilin interaction impairs cell steering. *Mol. Biol. Cell* **20**, 4509-4523 (2009).

8. Tsai FC, Meyer T. Ca2+ pulses control local cycles of lamellipodia retraction and adhesion along the front of migrating cells. *Curr. Biol.* **22**, 837-842 (2012).
9. Camley BA, Zhao Y, Li B, Levine H, Rappel WJ. Crawling and turning in a minimal reaction-diffusion cell motility model: Coupling cell shape and biochemistry. *Phys. Rev. E.* **95**, 012401 (2017).
10. Mogilner A, Rubinstein B. Actin disassembly 'clock' and membrane tension determine cell shape and turning: a mathematical model. *J. Phys. Condens. Matter.* **22,** 194118 (2010).
11. Gorelik R, Gautreau A. The Arp2/3 inhibitory protein arpin induces cell turning by pausing cell migration. Cytoskeleton (Hoboken). **72,** 362-71 (2015).
12. Lee CW1, Vitriol EA, Shim S, Wise AL, Velayutham RP, Zheng JQ. Dynamic localization of G-actin during membrane protrusion in neuronal motility. *Curr. Biol.* **23,** 1046-56 (2013).
13. Lee J, Ishihara A, Oxford G, Johnson B, Jacobson K. Regulation of cell movement is mediated by stretch-activated calcium channels. *Nature.* **400,** 382-386 (1999).
